# Supplementary material for: Dichotomy of heavy and light pairs of holes in the t−J model
Source: Nat Commun. 2023 Dec 4;14:8017. doi: 10.1038/s41467-023-43453-2 (PMC10695955; doi:10.1038/s41467-023-43453-2)
Supplement: Supplementary file 1 — Supplementary Information [file 41467_2023_43453_MOESM1_ESM.pdf]

# Supplementary Material: Dichotomy of heavy and light pairs of holes in the $t-J$ model

A. Bohrdt,[1, 2, *] E. Demler,[3] and F. Grusdt[4, 5]

[1]*ITAMP, Harvard-Smithsonian Center for Astrophysics, Cambridge, MA 02138, USA*
[2]*Department of Physics, Harvard University, Cambridge, Massachusetts 02138, USA*
[3]*Institut für Theoretische Physik, ETH Zurich, 8093 Zurich, Switzerland*
[4]*Department of Physics and Arnold Sommerfeld Center for Theoretical Physics (ASC), Ludwig-Maximilians-Universität München, Theresienstr. 37, München D-80333, Germany*
[5]*Munich Center for Quantum Science and Technology (MCQST), Schellingstr. 4, D-80799 München, Germany*

(Dated: November 7, 2023)

## I. COMPARISON TO STRING THEORY

In Supplementary Figs. 1, 2, and 3, we compare the prediction of the geometric string theory, [1], to the numerically calculated two-hole spectral function in the $t-J_z$ model at $t/J_z = 3$ for $s$-wave, $p$-wave, and $d$-wave pairs, respectively. Due to the finite evolution time in the MPS simulations, the corresponding spectra are broadened. The overall distribution of spectral weight agrees well between string theory and numerical $t-J$ model simulation for all values of $m_4$, and moreover, the dispersion of the peaks is in many cases correctly captured. As mentioned in the main text, the MPS simulations on the four-leg cylinder can lead to hybridization between $m_4 = 0$ and $m_4 = 2$, such that very weak remnants of $m_4 = 2$ states are visible in the $m_4 = 0$ spectra and vice versa. The geometric string theory does not suffer from such types of finite size effects, and no hybridization of different $m_4$ is possible.

## II. INTUITIVE PICTURE FOR FLAT BANDS OF $d$-WAVE PAIRS

As a nearest neighbor pair with $d$-wave symmetry moves through the system, the phase structure of the pair can lead to an exact cancellation of the final states. In Supplementary Fig. 4, such a hopping is depicted for an up/right movement of a pair of distinguishable particles connected by a string. The final configurations are the same for an initial $x$- and $y$-configuration of the nearest-neighbor pair, but due to the $d$-wave symmetry, the signs lead to a cancellation. This intuitive picture is valid in the perturbative regime, $t \ll J_z$, since the pair size is small and the hopping of the pair considered here is a higher order process. Our numerics presented in the main text and semi-analytical calculations presented in [1] indicate that this destructive interference is equally present beyond the perturbative regime $t \geq J_z$.

* Corresponding author email: annabelle.bohrdt@physik.uni-regensburg.de

## III. EXPERIMENTAL DETECTION OF PAIRS

To experimentally probe our predictions in solids, we discuss different possible approaches. The first method is to use coincidence angle-resolved photo-emission spectroscopy (cARPES) [2], which relies on simultaneous measurements of two photo-electrons and provides direct insights into the pair Green's function. As a main advantage of this approach it directly provides energy and momentum resolution across the entire Brillouin zone. A main disadvantage is that only $s$-wave pairs can be detected without further complications.

A second method assumes as a starting point an unpaired state of magnetic polarons around their dispersion minimum at momentum $(\pi/2, \pi/2)$. This state can either be assumed to be realized in equilibrium, or a previous ARPES pump pulse can create an initial occupation of magnetic polarons around $(\pi/2, \pi/2)$. Then a probe ARPES pulse is used to create pairs of holes directly from the initial magnetic polarons whose energy and momentum is known. To avoid an undesired signal from newly created magnetic polarons by the second ARPES beam, the ARPES signal without any prior doping can be subtracted. We envision that non-zero angular momentum channels $m_4 \neq 0$ can also be addressed using this method, if the initial magnetic polarons are created in a rotationally excited state [3] using a two-photon scheme involving a driven phonon mode carrying non-zero $C_4$ angular momentum $m_4$ [4].

A third approach is based on Anderson-Goldman pair tunneling in a tunnel junction setup [5]: to study the structure of individual pairs in a strongly underdoped quasi-2D material as considered here, we propose to tunnel-couple the latter to a probe-superconductor along $z$ direction. By applying an in-plane magnetic field $H_y$ along $y$ and a voltage $V$ across the junction, Cooper pair tunneling from the physical to the probe layer can be described by an effective Hamiltonian of the form [6, 7]

$$\hat{\mathcal{H}} = -g \int dxdy \; e^{i(qx-\omega t)} \Delta_p(x,y) \hat{\Delta}^{(s)}_{m_4}(x,y) + \text{h.c.} \quad (1)$$

Here $\Delta_p(x,y)$ is the order parameter in the probe-superconductor, which is assumed to be well below its critical temperature $T \ll T_c$. By choosing different singlet superconductors as probes, situations with $s$- or $d$-

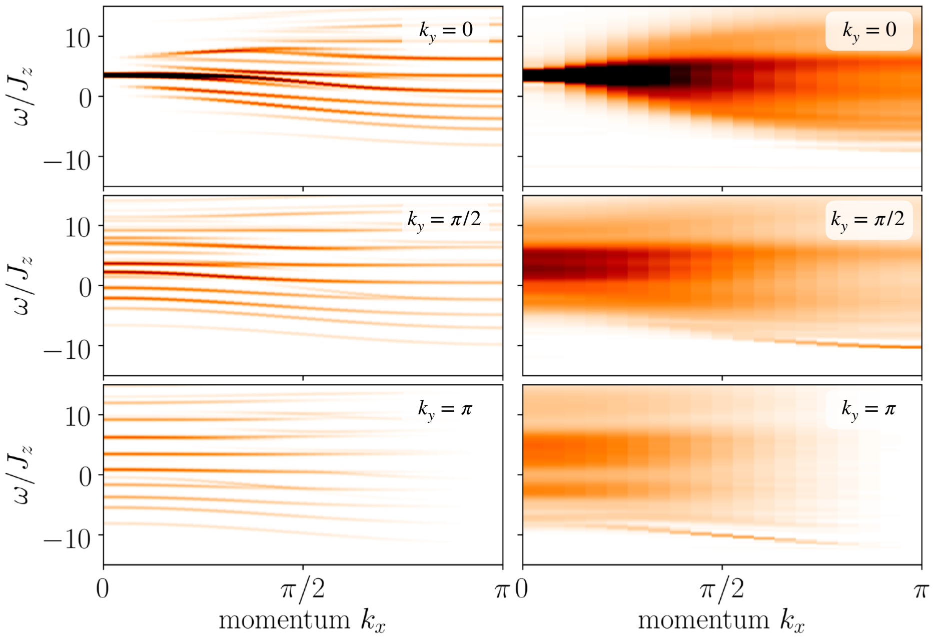


Supplementary Figure 1. **Comparison of $s$-wave spectra to geometric string theory** prediction, [1], left, to numerically calculated spectra (right), for the $t-J_z$ model with $t/J_z = 3$, $\chi = 600$, time evolution up to $T_{max}/J_z = 10$, on a $40 \times 4$ cylinder. Top, middle, bottom plots correspond to momentum $k_y = 0, \pi/2, \pi$, respectively.

wave pairing symmetry ($m_4 = 0$ or $m_4 = 2$) could be realized. In the physical system this introduces the proximity-coupling term $\hat{\Delta}^{(s)}_{m_4}(x, y)$ introduced previously, with the same pairing symmetry as in the probe. The coupling strength $g \propto t_{\rm sp}^2$ is proportional to the weak tunnel coupling $t_{\rm sp}$ of the junction.

The in-plane momentum $q$ along $x$, i.e in the plane of the junction and perpendicular to the magnetic field, can be tuned by $H_y$ [7]

$$q = \frac{2eH_y}{\hbar c}\,(\lambda + d/2) \qquad (2)$$

with $\lambda$ the magnetic penetration depth of the probe-superconductor and $d$ the thickness of the physical sample where the pairs are created. The frequency $\omega = 2eV/\hbar$ is controlled by the voltage across the junction.

In principle this setup allows to measure the frequency and momentum resolved tunnel-current $I(\omega, \boldsymbol{q})$ across the junction, which is expected to exhibit peaks when (meta-) stable paired states of holes are created in the sample. The positions of these peaks reveal the two-hole spectra we predicted in our article. The main drawback of the tunnel-junction approach is the limited range of momenta $q \ll 1/a$, where $a$ is the lattice constant, that can be accessed by realistic magnetic fields. However, provided enough frequency resolution can be achieved, exploring the small-$q$ regime would be sufficient to measure the effective mass of the hole pairs.

In ultracold atomic systems similar spectroscopic methods can be implemented by tunneling into a second probe system [8, 9]. By considering two-particle hoppings and paired final states, the desired pair Green's function can be measured in such settings. Moreover, by combining them with additional lattice modulations, the angular momentum of the pairs can be modified [4].

## IV. MPS SIMULATIONS

In order to obtain the spectral functions shown in the main text, we follow the same procedure described in detail in [10] and [3]. In the following, we discuss the procedure as well as different convergence checks in more detail.

### A. Convergence

We calculate the real-time and -space rotational Green's function based on the singlet pair operator, Eq.(3) of the main text, using time-dependent matrix product states. The time evolution used for the rotational spectra shown in the main text is calculated using the $W^{(II)}$ method [11] with a time step of $dt = 0.02/J$. The resulting real-space and real-time Green's function is shown in Supplementary Fig. 5 for different bond dimensions $\chi$ (increasing opacity corresponds to increasing bond dimension) for two exemplary distances.

We then perform a Fourier transform to momentum space to obtain $\mathcal{G}^{(m_4)}_{\rm rot}(\boldsymbol{k}, t)$, Eq.(5) of the main text. Subsequently, perform a linear prediction in order to ex-

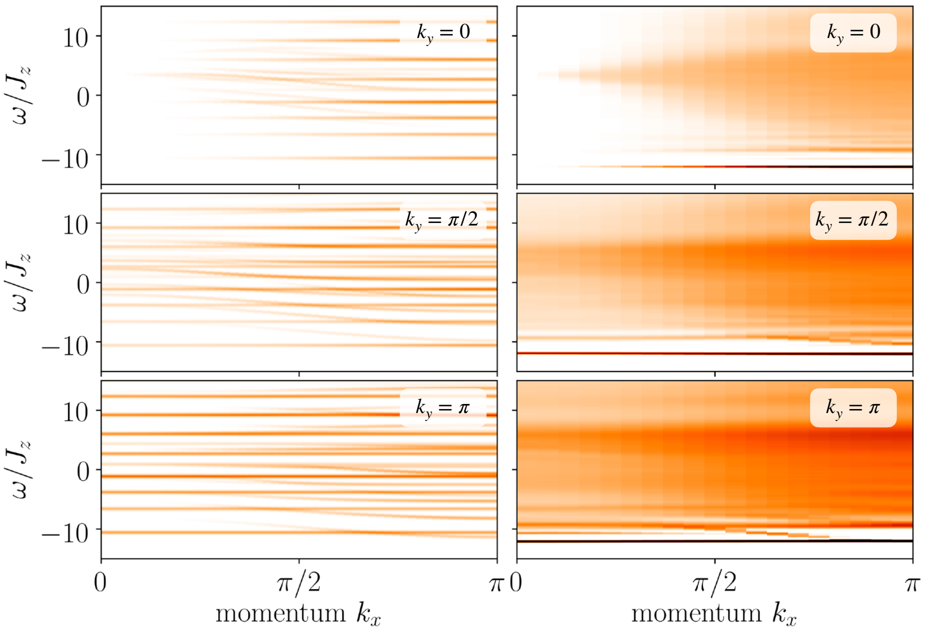


Supplementary Figure 2. **Comparison of $p$-wave spectra to geometric string theory** prediction, [1], left, to numerically calculated spectra (right), for the $t - J_z$ model with $t/J_z = 3$, $\chi = 600$, time evolution up to $T_{max}/J_z = 10$, on a $40 \times 4$ cylinder. Top, middle, bottom plots correspond to momentum $k_y = 0, \pi/2, \pi$, respectively.

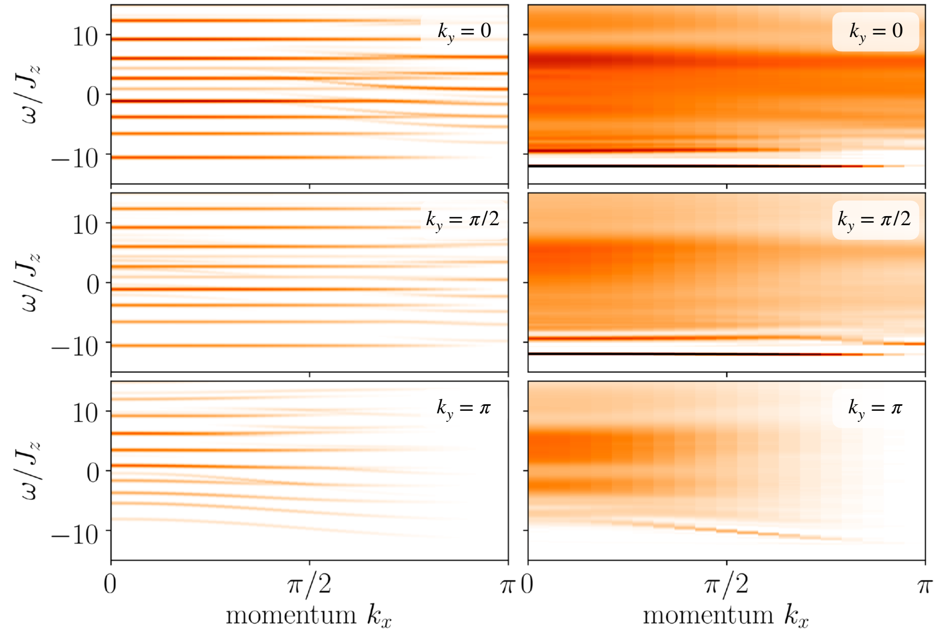


Supplementary Figure 3. **Comparison of $d$-wave spectra to geometric string theory** prediction, [1], left, to numerically calculated spectra (right), for the $t - J_z$ model with $t/J_z = 3$, $\chi = 600$, time evolution up to $T_{max}/J_z = 10$, on a $40 \times 4$ cylinder. Top, middle, bottom plots correspond to momentum $k_y = 0, \pi/2, \pi$, respectively.

tend our time signal beyond the computationally accessible regime [12]. Finally, the time signal is multiplied by a Gaussian envelope $w(t) = \exp\left[-0.5(t\sigma_\omega)^2\right]$, where $\sigma_\omega = \sigma/T_{\max}$. The Fourier transformed signal, i.e. the resulting spectral function, is shown for different choices of the width of the Gaussian envelope in Supplementary Fig. 6.

As can be seen in Supplementary Fig. 6, the finite evolution time leads to some artifacts in the Fourier transformed signal, in particular some negative values of the

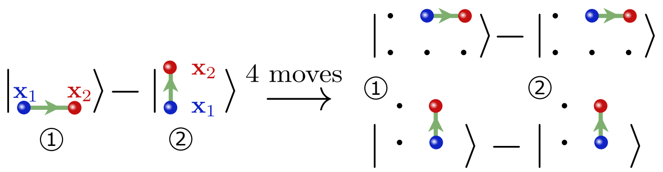


Supplementary Figure 4. **Intuitive picture** for the appearance of flat bands for $d$-wave pairs.

spectral function. Increasing $\sigma_\omega$ leads to broader peaks, but also suppresses the sharp cutoff from the finite evolution time more effectively, thus reducing the un-physical negative signal.

### B. Extraction of peak position

We extract the positions of the peaks shown in Figs.3 and 4 of the main text by determining the lowest value

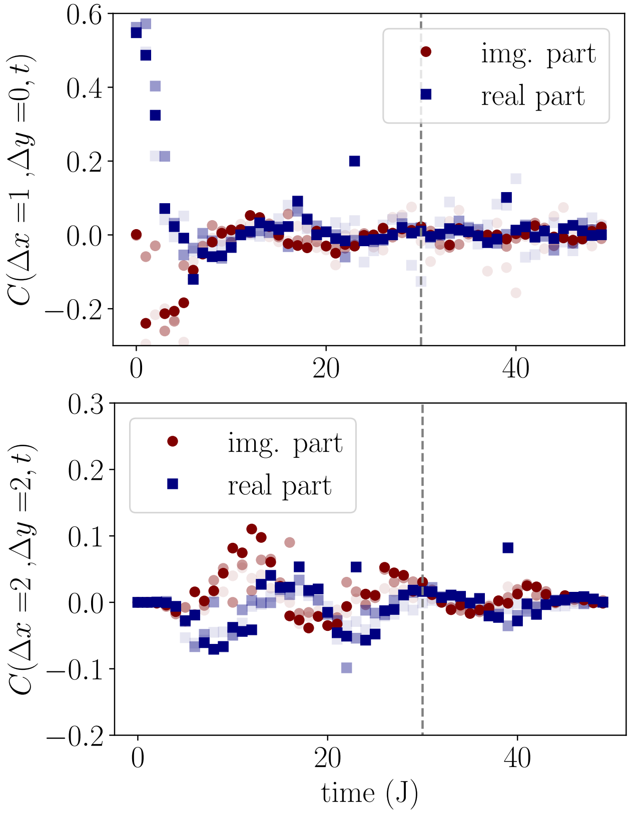


Supplementary Figure 5. **Convergence with bond dimension.** Real-space and -time Green's function at exemplary distances $\Delta x$, $\Delta y$ (top and bottom) for bond dimensions $\chi = 600, 1200, 2000$ (increasing opacity) for the $t-J$ model. The rotational spectra shown in the main text and supplementary material are based on the time evolution up to $T_{\max} = 30$ (gray dashed line).

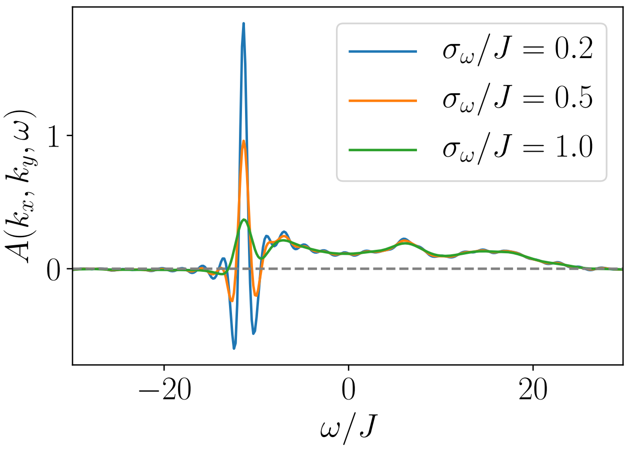


Supplementary Figure 6. **Gaussian envelope.** Spectral function at $k_x = 0, k_y = 0$, $m_4 = 2$ for different widths $\sigma_\omega$ of the Gaussian envelope for the $t-J$ model with $t/J = 3$, $\chi = 1200$. The rotational spectra shown in the main text and supplementary material are based on the time evolution up to $T_{\max} = 30$ and Gaussian envelope width $\sigma_\omega/J = 0.5$.

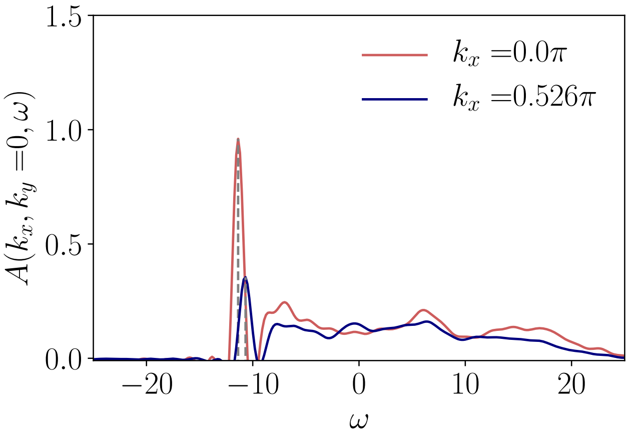


Supplementary Figure 7. **Extracting peak positions** from the spectral function, here shown for $k_y = 0$ and $k_x$ as indicated for the $t-J$ model with $t/J = 3$, $\chi = 1200$ and $m_4 = 2$. Peak positions shown in main text Figs. 3 and 4 correspond to dashed gray lines in this plot.

of $\omega$ at which $A(\boldsymbol{k}, \omega^* - \Delta\omega) < A(\boldsymbol{k}, \omega^*)$ and $A(\boldsymbol{k}, \omega^* + \Delta\omega) > A(\boldsymbol{k}, \omega^*)$, where $\Delta\omega$ is the resolution in frequency space. We set a minimum height of the peak $A(\boldsymbol{k}, \omega^*) \geq 0.05$ in order to discard artifacts of the Fourier transform.

### C. Singlet versus triplet excitations

In Supplementary Fig. 8, the rotational two-hole spectrum is compared for the case of a singlet excitation, Eq.(4) of the main text, and a triplet excitation, Eq.(9) of the main text, for the $t-J$ model. As expected, the overall amplitude of the triplet excitation is significantly

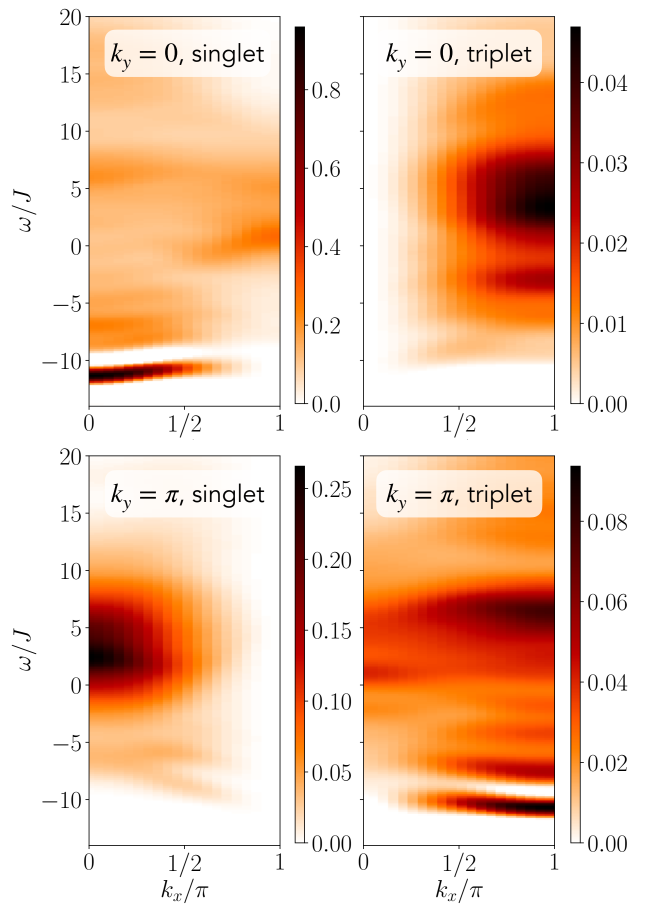


Supplementary Figure 8. **Singlet vs triplet excitation.** Rotational two-hole spectra in the $t-J$ model with $t/J = 3$ for $m_4 = 2$ ($d$-wave) and $k_y = 0$ (top) and $k_y = \pi$ (bottom) for singlet (left) and triplet (right) excitation, obtained from time-dependent matrix product state simulations of the singlet two-hole rotational spectrum.

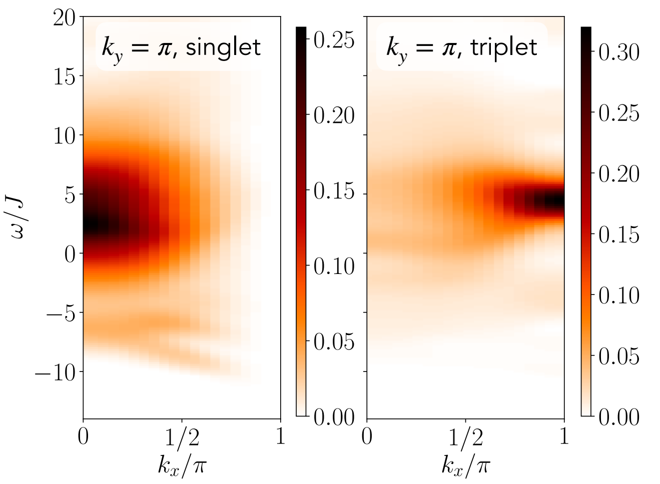


Supplementary Figure 9. **Singlet vs triplet excitation.** Rotational two-hole spectra in the $t-J$ model with $t/J = 3$ for $m_4 = 0$ ($s$-wave) and $k_y = \pi$ for singlet (left) and triplet (right) excitation, obtained from time-dependent matrix product state simulations of the singlet two-hole rotational spectrum.

lower than in the case of a singlet excitation; note the different color scale. Moreover, the lowest energy peak for the triplet excitation is at a higher energy than the corresponding lowest energy peak in the singlet excitation spectrum.

The triplet excitation spectra at momentum $\boldsymbol{k} = (\pi, \pi)$ is related to the dynamical correlation functions involving $\pi^\dagger_{s/d}$ operators considered in [13]. Consistent with this earlier work on finite size Fermi-Hubbard models, a low energy peak with high spectral weight is visible in the $d$-wave spectra. In the $s$-wave case, we find the majority of spectral weight at high energies.

## V. DIFFERENT ANGULAR MOMENTA

In Supplementary Fig. 10, the rotational two-hole spectra are shown for $m_4 = 0, 1, 2$ and $k_y = 0, \pi/2, \pi$ for the $t-J$ model.

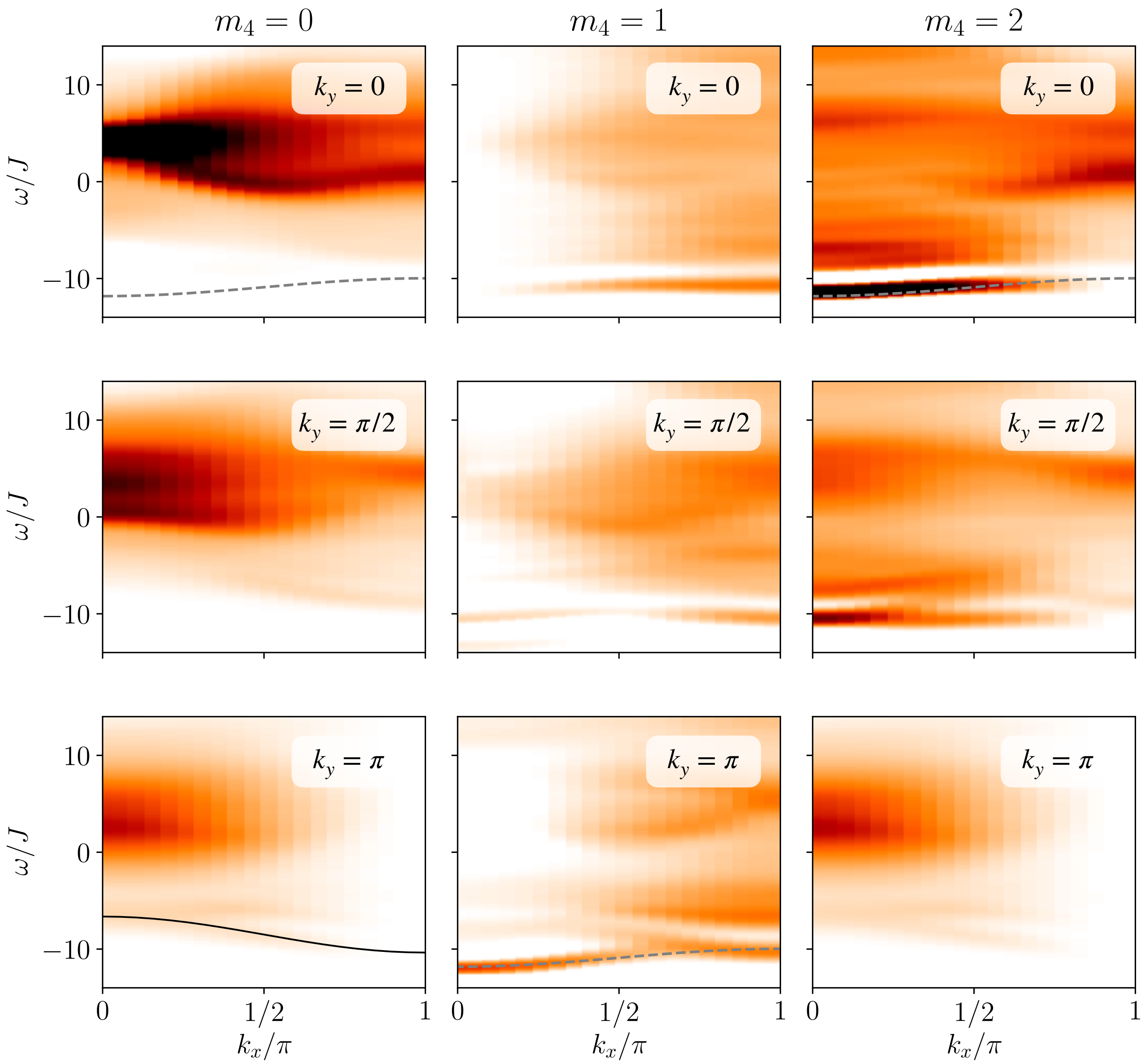


Supplementary Figure 10. **Angular momentum dependence** of the rotational two-hole spectra in the $t-J$ model with $t/J = 3$ for $m_4 = 0, 1, 2$ (left, middle, right column) and $k_y = 0, \pi/2, \pi$ (top, middle, bottom row). The colormap corresponds to matrix product state simulations of the singlet two-hole rotational spectrum. The black line corresponds to the cosine fit to the lowest dispersive feature at $k_y = \pi$, see Fig.3 of the main text. The gray dashed lines correspond to the cosine fit to the lowest peak at $k_y = 0$, see Fig.4 of the main text.

## REFERENCES

[1] Fabian Grusdt, Eugene Demler, and Annabelle Bohrdt. Pairing of holes by confining strings in antiferromagnets. *SciPost Phys.*, 14:090, 2023.

[2] Yuehua Su and Chao Zhang. Coincidence angle-resolved photoemission spectroscopy: Proposal for detection of two-particle correlations. *Phys. Rev. B*, 101:205110, May 2020.

[3] A. Bohrdt, E. Demler, and F. Grusdt. Rotational resonances and regge-like trajectories in lightly doped antiferromagnets. *Phys. Rev. Lett.*, 127:197004, Nov 2021.

[4] A. Bohrdt, E. Demler, and F. Grusdt. Rotational resonances and regge trajectories in lightly doped antiferromagnets. *arxiv:2101.09280*, 2021.

[5] J. T. Anderson, R. V. Carlson, and A. M. Goldman. Pair tunneling as a probe of order-parameter fluctuations in superconductors: Zero magnetic field effects. *Journal of Low Temperature Physics*, 8(1):29–46, 1972.

[6] P. W. Anderson and C. J. Gorter. Chapter i the josephson effect and quantum coherence measurements in superconductors and superfluids. In *Progress in Low Temperature Physics*, volume 5, pages 1–43. Elsevier, 1967.

[7] D. J. Scalapino. Pair tunneling as a probe of fluctuations in superconductors. *Phys. Rev. Lett.*, 24:1052–1055, May 1970.

[8] A. Bohrdt, D. Greif, E. Demler, M. Knap, and F. Grusdt. Angle-resolved photoemission spectroscopy with quantum gas microscopes. *Phys. Rev. B*, 97:125117, Mar 2018.

[9] Peter T. Brown, Elmer Guardado-Sanchez, Benjamin M. Spar, Edwin W. Huang, Thomas P. Devereaux, and Waseem S. Bakr. Angle-resolved photoemission spectroscopy of a fermi–hubbard system. *Nature Physics*, 16(1):26–31, Oct 2019.

[10] Annabelle Bohrdt, Eugene Demler, Frank Pollmann, Michael Knap, and Fabian Grusdt. Parton theory of angle-resolved photoemission spectroscopy spectra in antiferromagnetic mott insulators. *Phys. Rev. B*, 102:035139, Jul 2020.

[11] Michael P. Zaletel, Roger S. K. Mong, Christoph Karrasch, Joel E. Moore, and Frank Pollmann. Time-evolving a matrix product state with long-ranged interactions. *Phys. Rev. B*, 91:165112, Apr 2015.

[12] Thomas Barthel, Ulrich Schollwöck, and Steven R. White. Spectral functions in one-dimensional quantum systems at finite temperature using the density matrix renormalization group. *Phys. Rev. B*, 79:245101, Jun 2009.

[13] Stefan Meixner, Werner Hanke, Eugene Demler, and Shou-Cheng Zhang. Finite-size studies on the so(5) symmetry of the hubbard model. *Phys. Rev. Lett.*, 79:4902–4905, Dec 1997.
